# Energy Intake and Appetite Sensations Responses to Aquatic Cycling in Healthy Women: The WatHealth Study

**DOI:** 10.3390/nu13041051

**Published:** 2021-03-24

**Authors:** Lore Metz, Laurie Isacco, Nicole Fearnbach, Bruno Pereira, David Thivel, Martine Duclos

**Affiliations:** 1 Laboratory of the Metabolic Adaptations to Exercise under Physiological and Pathological Conditions, (AME2P), UE3533, Clermont Auvergne University, CEDEX 63170 Aubiere, France; laurie.isacco@uca.fr (L.I.); david.thivel@uca.fr (D.T.); 2 Auvergne Research Center for Human Nutrition (CRNH), CEDEX 63000 Clermont-Ferrand, France; 3 Pennington Biomedical Research Center, Baton Rouge, LA 70808, USA; Nicole.fearnbach@pbrc.edu; 4 Clermont-Ferrand University Hospital, Biostatistics Unit (DRCI), CEDEX 63000 Clermont-Ferrand, France; bpereira@chu-clermontferrand.fr; 5 Department of Sport Medicine and Functional Explorations, Clermont-Ferrand University Hospital, G. Montpied Hospital, CEDEX 63000 Clermont-Ferrand, France; mduclos@chu-clermontferrand.fr; 6 Unité Mixte de Recherche 1019, INRAe, UMR 1019, CEDEX 63000 Clermont-Ferrand, France

**Keywords:** immersed exercise, appetite, energy intake, energy expenditure

## Abstract

Background: The aim of this study was to investigate energy expenditure, food intake and appetite feelings in response to water- vs. land-based cycling exercises in healthy young women. Methods: Anthropometric measurements and body composition were assessed among 20 women who performed four experimental sessions in a randomized order: (i) a rest condition (CONT); (ii) a 30-min aqua-cycling exercise session (WAT), (iii) a 30-min land-cycling exercise session at the same rpm (LAND), (iv) a land-cycling session at the same heart rate and isoenergetic to WAT (LAND-Iso). Energy expenditure and substrate oxidation were measured by indirect calorimetry; ad libitum energy intake during subsequent lunch was assessed with appetite feelings recorded at regular intervals. Results: Energy expenditure was higher during the 30-min WAT than during CONT and LAND (*p* < 0.001). Carbohydrate oxidation was higher in the WAT session compared to CONT and LAND (*p* < 0.05). LAND-Iso duration was significantly increased (+14 min) to reach the same energy expenditure as in the WAT condition (*p* < 0.05). There was no differences in food intake between sessions. Conclusion: While further studies are needed to optimize the chronic energetic effects of aqua-cycling, the present study suggests that this exercise modality could represent an efficient strategy to induce acute energy deficit.

## 1. Introduction

Public health policies promote healthy active living to prevent the development of chronic diseases that have been shown to be associated with inactivity [1]. Healthy lifestyles mainly rely on an optimal control of energy balance through both energy expenditure and intake [2]. The popularity of water-based activities, mainly aqua-cycling, has shown an impressive progression for the last couple of years, especially in women who are looking for weight control and weight management. A recent systematic review from Rewald et al. [3] showed that studies comparing land- vs. water-based exercises mainly focused on cardiovascular adaptations during protocols for maximal aerobic capacity testing. Brechat et al. [4] have studied the metabolic adaptations to water-cycling exercise in healthy young men, showing a 25% increased oxygen consumption during water compared with land exercise. The potential efficiency of water immersion exercise to impact both sides of the energy balance equation has not been clearly addressed, and is essential to prescribe optimal weight management programs. Data on specific metabolic adaptations in women are still needed, as well as investigations of the potential subsequent food intake responses to exercise. Indeed, while energy intake (EI) and energy expenditure (EE) have been long considered as independently influencing energy balance, a growing body of literature suggests that they may be coupled with exercise having indirect effects on energy consumption and appetite control [5,6]. Few studies have investigated food intake and appetite feelings in response to water-based exercise. In one study, White and collaborators showed that a 45-min imposed cycling exercise set at moderate intensity (60% of VO_2max_) favored increased subsequent food intake when performed in the cold compared with resting condition and thermoneutral water temperature (20 vs. 33 °C) [7]. Interestingly, EI was not altered when the same exercise was completed in 33 °C or in 20 °C water [7]. More recently, Ueda and collaborators asked healthy men to cycle for 30 min at 50% of their maximal aerobic capacities once land-based and once immersed (34 °C water) [8]. In this study, hunger was lower in response to the water-based trial, without any difference in absolute postexercise EI between conditions [8]. More recently, Thackray and collaborators [9] showed that 60 min of swimming increased subsequent EI compared to cycling exercise and a control session. However, the different nature of these exercise modalities makes it difficult to understand the effect of immersion per se during exercise on EI. Importantly, all of these studies were conducted among healthy young men, while current aqua-cycling programs almost exclusively serve women. Moreover, no study has yet addressed the effect of acute aqua-cycling exercise on both components of energy balance.

Therefore, the aim of this study was to investigate energy expenditure, food intake and appetite sensations in response to water- vs. land-based cycling exercises in healthy young women. We compared aqua-cycling exercise to a land-based cycle exercise set at the same absolute intensity, as well as an isoenergetic land-based cycling session set at the same relative intensity. We hypothesized that an acute water-based exercise would favor energy expenditure and reduce food intake compared with the land-based condition.

## 2. Materials and Methods

Twenty young women were recruited through advertisements and took part in a screening session to ensure that they met the following inclusion criteria: age between 18 and 40 years, not pregnant, free of any disease or food allergies, weight stable for ≥6 months before their enrollment in the study (±2 kg), not following a special diet or taking any medications except oral contraceptive (10 participants were taking combined oral contraceptives) that could influence EE or food intake. All women had a low habitual physical activity level, with fewer than 2 h per week of moderate physical activity, as indicated by the International Physical Activity Questionnaire (IPAQ). Informed consent was obtained from all participants. This study was approved by the local ethical committee (CPP Sud Est VI, AU-1247) and registered as a clinical trial (NCT 02895217).

### 2.1. Design 

**Figure 1:**
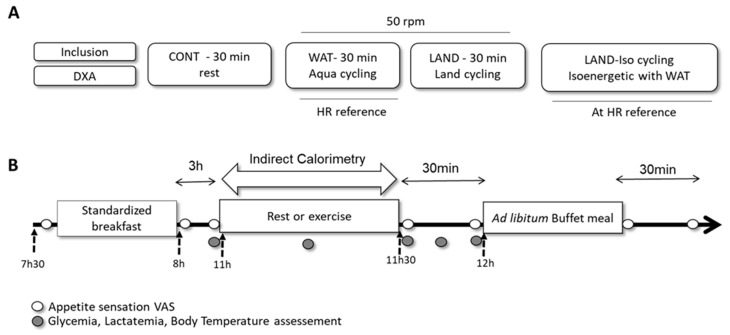
Schematic representation of the study design (**A**), and of experimental session (**B**). DXA: Dual X-ray Absorptiometry; CONT: control session; WAT: Water cycling session; LAND: Land cycling session.

After a full medical examination to assess eligibility, the included participants were asked to complete a food preference questionnaire (which was used to compose the buffet meals presented during the experimental sessions). Anthropometric measurements were taken and body composition was assessed by dual-energy X-ray absorptiometry (DXA). They were then asked to complete four experimental sessions performed in a semirandomized order and separated by at least seven days: (i) a control session where they remain at rest for 30 min (CONT); (ii) an exercise session of aqua-cycling for 30 min at 50 rpm (WAT), (iii) an exercise session of land-based cycling for 30 min at 50 rpm (LAND), (iv) an exercise session of land-based cycling at the same mean heart rate (HR) and iso-energetic to the WAT session (LAND-Iso) with no predetermined duration. The general design of the protocol and experimental session detailed are presented in Figure 1A,B, respectively. Participants were asked to avoid exercise training the day before each evaluation session and to maintain their habitual level of physical activity during the entire study period. They were also asked to avoid smoking and caffeine consumption on the morning of each session. Energy expenditure was measured by indirect calorimetry during each exercise session; subsequent EI at lunch was assessed and appetite feelings were measured at regular intervals. The participants were asked to rate their perceived exertion during the exercise sessions. Glycaemia and lactatemia and tympanic temperature were also assessed during each session, described in more detail below. 

### 2.2. Anthropometrics and Body Composition Measurements

A digital scale (Seca, Les Mureaux, France) was used to measure body weight to the nearest 0.1 kg, and barefoot standing height was assessed to the nearest 0.1 cm using a wall-mounted stadiometer (Seca, Les Mureaux, France). Body mass index (BMI) was calculated as body weight (kg) divided by height squared (m^2^). Fat mass (FM) and fat-free mass (FFM) were assessed using DXA following standardized procedures (QDR4500A scanner, Hologic, Waltham, MA, USA). These measurements were obtained during the preliminary visit by a trained technician.

### 2.3. Energy Intake Assessment

At 8:30 am, a standardized breakfast was offered (same composition and caloric content as previously detailed) [10]. Thirty minute after rest (CONT condition) or the exercise sessions (WAT, LAND and LAND-Iso), an ad libitum buffet-type lunch was offered to the participants based on their preferences as determined by the food questionnaire completed during the preliminary visit [11]. Top rated items were avoided to limit overconsumption and items indicated as “liked but rarely consumed” were not provided to avoid occasional eating. Participants were provided with an ad libitum buffet meal for lunch (12:00 p.m.). Food consumption was weighed and recorded by investigators (Bilnut 4.0 SCDA Nutrisoft software, France) to calculate total EI during lunch. The proportion of the total EI derived from fat, carbohydrate and protein was calculated using the same nutritional software. Relative energy intake (REI) was then calculated as REI = EI − EE, for each condition. 

### 2.4. Subjective Appetite Sensations

At regular intervals throughout the day from 8:00 a.m. to 30 min after lunch, participants were asked to rate their hunger, satiety and desire to eat using visual analogue scales (VAS of 100 mm), for which reliability has been previously reported [12]. Participants completed VAS before and after breakfast, immediately before and after rest/exercise sessions, immediately before and after the ad libitum lunch as well as 30 min after lunch. The satiety quotient (SQ), a marker of an individual’s satiation efficiency, was calculated for lunch using hunger (Hunger SQ) and satiety (Satiety SQ) ratings as follows [13]:

Satiety quotient mm/kcal = [(premeal appetite sensation mm) − (30 min postmeal VAS mm))/energy content of the meal (kcal)] × 100

### 2.5. Metabolic and Cardiorespiratory Parameters

After calibration following manufacturer’s recommendation before each session, oxygen consumption (VO_2_), carbon dioxide production (VCO_2_), ventilation (VE) and HR were continuously recorded throughout each session using indirect calorimetry (K4b^2^, Cosmed, Rome, Italy) and HR monitor (Polar V800, Kempele, Finland). Total EE over the session was calculated as follows: VO_2_ (L min^−1^) × energy equivalent of oxygen × duration (min). Respiratory exchange ratio (RER; VCO_2_/VO_2_) and carbohydrate (CHO) and lipid oxidation rates were calculated at rest and over the entire period of each session: CHO = 4.585VCO_2_ − 3.2255VO_2_Lipid = 1.6946VO_2_ − 1.7012VCO_2_ where CHO and lipid are in g min^−1^, and VCO_2_ and VO_2_ are in L min^−1^ [14].

### 2.6. Rate of Perceived Exertion

During each exercise session, at 15 min and the end of exercise, the RPE was measured using the 6- to 20-point Borg scale, where 6 means “no exertion at all” and 20 means maximal exertion [15]. During the screening visit, the range of sensations that correspond to effort categories within the Borg scale were explained to the participants to familiarize them with it.

### 2.7. Glycemia, Lactatemia and Body Temperature

Glycemia and lactatemia were measured from fingertip capillary blood samples before (T0), at 15 min (T15) and at the end of exercise or rest session (end) and at 15 (15 min rec) and 30 (30 min rec) in recovery using Accu-Chek Performa (Roche Diagnostics, Penzberg, Germany) and Lactate Pro 2 (Arkray, KDK Corporation, Minami-Ku, Kyoto, Japan) devices, respectively. In addition, tympanic temperature was assessed at the same time points (Braun GmbH, Thermoscan 3, Kronberg; Germany).

### 2.8. Description of the Experimental Sessions 

Control session (CONT): from 11:15 a.m. to 11:45 a.m., the participants remained seated on a comfortable chair (30 min). They were not allowed to talk, read, watch TV or to complete any intellectual tasks. They were equipped with an indirect calorimeter (K4b2 COSMED Inc, Pavona, Italy) to measure their resting EE and HR was continuously recorded (Polar technology monitor).

Aqua-cycling session (WAT): from 11:15 a.m. to 11:45 a.m., the participants were invited to perform an aqua-cycling exercise in a 27 °C water, using a specific aqua-bike technology (Hydrorider^®^ Aquabike Professional, San Lazzaro di Savena, Italy). The participants were asked to cycle at a fixed 50 revolution per minute (rpm), following a metronome. This rpm has been identified as comfortable during cycling in water [16]. This device does not have any brake system, resistance or drag forces depend on rate of motion per minute. The participants were asked to rate their perceived exertion [15] at 15 min and at the end of the exercise. 

Land session (LAND): the experimental session was similar that previously described for WAT session (30 min—50 rpm) except that the exercise took place in an ordinary room at neutral temperature (21 °C). The same bike was used for this exercise session allowing the investigation of all parameters without the effect of water drag forces.

Land-Iso session (LAND-Iso): Exercise took place on the same device as WAT and LAND sessions in a room at neutral temperature (21 °C). The participants were asked to reach, within 2 min, the mean HR obtained during the WAT session and to maintain it during the whole session. The session for each subject was stopped when EE reached similar values that for WAT session. Time needed to reach similar amount of EE than for WAT session and mean rpm were recorded for each subject. 

### 2.9. Statistical Analysis

Analyses were performed using Statview 5.0 (SAS Institute, Cary, USA). Results are expressed as mean ± standard deviation). The sample size estimation was determined according to data reported in the literature [17,18] and to Cohen’s recommendations who has defined effect-size bounds as: small (ES: 0.2), medium (ES: 0.5) and large (ES: 0.8, “‘grossly perceptible and therefore large”). Effect size for ANOVA was calculated with partial eta square. The distribution of the data was tested using the Smirnov–Kolmogorov test. One-way ANOVA were used to compare energy intake, macronutrient consumption as well as energy expenditure and relative energy intake between the different experimental conditions. Repeated-measures ANOVA were used to compare appetite feelings Area under the Curve (AUC), glycemia, lactatemia and tympanic temperature between conditions. Spearman correlations were performed between perceived exertion, FM (%), FFM (kg), energy expenditure and the absolute and relative energy intake. The level of significance was set at *p* < 0.05. 

## 3. Results

The subjects’ characteristics concerning age, anthropometric and body composition parameters are presented in Table 1.

### 3.1. Energy Expenditure and Substrate Utilization

**Figure 2:**
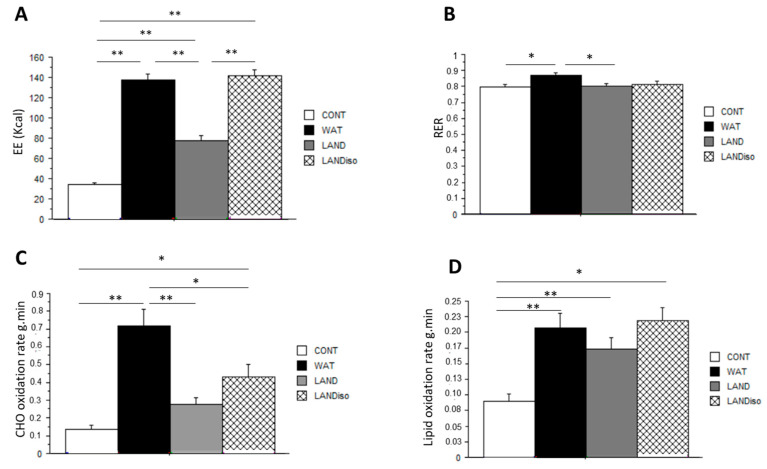
Energy expenditure and substrate utilization rates. (**A**) Energy expenditure (EE); (**B**) respiratory exchange ratio (RER), (**C**) carbohydrate oxidation rates and (**D**) lipid oxidation rates are presented for control (CONT), water-cycling (WAT), land-cycling (LAND) and land isoenergetic cycling (LAND-Iso) session. Data presented as mean ± SD; post hoc pairwise comparisons using Scheffe test were performed. *, **: significantly different at *p* < 0.05 and *p* < 0.01, respectively.

The three exercise sessions induced a significant increase in EE compared to the control session (CONT 34.2 ± 5.8 vs. WAT 137.2 ± 26.6 vs. LAND 77.4 ± 21.3 vs. LAND-Iso138 ± 26.1 kcal; *p* < 0.001, ES: 0.82) as shown in Figure 2A. As LAND-Iso was set to be iso-energetic to WAT session, there was no difference in EE between the two sessions, but they both induced a higher EE compared to the LAND condition (*p* < 0.05). Respiratory exchange ratio (RER) was higher in WAT compared to CONT and LAND (0.86 ± 0.08 vs. 0.79 ± 0.08 vs. 0.79 ± 0.07 *p* < 0.05, ES: 0.16). In line with this result, rate of carbohydrate oxidation was higher in WAT compared with the other sessions (0.14 ± 0.08 vs. 0.71 ± 0.4 vs. 0.27 ± 0.16 vs. 0.43 ± 0.3 g.min^−1^; *p* < 0.05; ES: 0.45). LAND-Iso also showed a higher rate of CHO oxidation compared with CONT session (*p* < 0.05). Lipid oxidation rate was higher in all exercise sessions compared with CONT session (0.08 ± 0.05 vs. 0.2 ± 0.1 vs. 0.17 ± 0.08 vs. 0.2 ± 0.09 g.min^−1^; *p* < 0.05; ES: 0.33). Energy expenditure and substrate utilization during the different sessions are depicted in Figure 2.

### 3.2. Cardiorespiratory Parameters and Perceived Exertion

Exercise sessions increased VO_2_ (ES: 0.87; *p* < 0.05) and VE (ES: 0.78; *p* < 0.05) compared with CONT session (Table 2). WAT session induced a higher increase in VO_2_ and VE compared with LAND and LAND-Iso sessions (*p* < 0.05). LAND-Iso session showed a higher VO_2_ and VE than LAND session (*p* < 0.05). Heart rate during exercise sessions was significantly increased compared to CONT condition (*p* = 0.89). Heart rate during LAND session was significantly lower than during the WAT and LAND-Iso sessions (ES: 0.78; *p* < 0.05). Session duration was significantly higher during the LAND-Iso exercise (ES: 0.75; *p* < 0.001) as shown in Table 2. Cadence in LAND-Iso was higher than in WAT session (71.7 ± 8.6 vs. 50 rpm; *p* < 0.001), this is logically explained by the need to increase rpm in absence of water drag force, to reach the same HR intensity than in WAT session. 

### 3.3. Food Intake and Appetite Sensations

**Figure 3:**
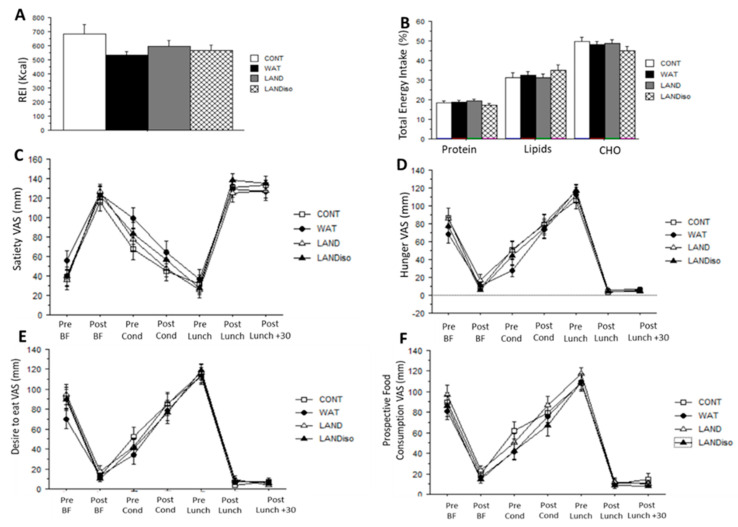
Food intake and appetite sensations. (**A**) Relative energy intake (REI), (**B**) macronutrients in percentage of total energy intake, (**C**) satiety feelings, (**D**) hunger feelings, (**E**) desire to eat feelings, (**F**) prospective food consumption, are presented for control (CONT), water-cycling (WAT), land-cycling (LAND) and land isoenergetic cycling (LAND-Iso) sessions. Data are presented as mean ± SD, significant difference is set at *p* < 0.05.

Total ad libitum EI at the buffet meal did not differ between conditions (714 ± 280 vs. 664 ± 135 vs. 673 ± 183 vs. 710 ± 151 kcal; ES: 0.01 *p* = 0.38) neither did REI (682 ± 288 vs. 531 ± 127 vs. 597 ± 172 vs. 567 ± 148 kcal; ES: 0.1 *p* = 0.45) (Figure 3A). Relative to CONT session (100%), the three exercise sessions induced a decrease of −23% (WAT), −13% (LAND), −17% (LAND-Iso) in REI. There was no difference in the macronutrient consumption between sessions (CHO; ES: 0.01; lipids ES: 0.03; protein ES: 0.01 *p* = 0.36) (Figure 3B). 

In regard to appetite sensations, there was a main effect of time for all the sessions (ES: 0.9 *p* < 0.05) but no condition or interaction effect. There was no significant difference in appetite feelings between sessions (ES: 0.01, *p* = 058) (Figure 3C–F) and no difference in AUC (Table 2). Similarly, no difference was found between sessions for SQ for satiety (ES: 0.02; *p* = 0.61) and hunger (ES: 0.01, *p* = 0.34) (Table 2).

### 3.4. Blood Parameters and Body Temperature 

There was no effect of time or condition for lactatemia, as shown in Table 3. There was a main effect of time for glycaemia and tympanic temperature (*p* < 0.05) (Table 3). The lowest glycaemia values were observed during WAT session, but this was not significantly different from the other conditions.

## 4. Discussion

To our knowledge, the present study is the first to investigate the effect of acute aquatic cycling on energy balance in healthy young women. We hypothesized that an acute water-based exercise would favor energy expenditure and reduce food intake compared with the land-based condition. Our results show that when performed at the same relative intensity (i.e., heart rate), 13.6 extra min are needed during a land-based cycling exercise to reach similar EE compared with a water-based cycling session. While lower values in EI and REI were observed during WAT session, it did not reach statistical significance.

The effect of aquatic immersion during cycling exercise on EE has not been thoroughly studied. Only Brechat et al. [4] have compared cardiovascular and respiratory responses, in young men, between land- vs. aquatic-exercise cycling sessions (60% of VO_2max_ for 30 min). Given that they focused on mechanical and metabolic power (watts and O_2_ consumption, respectively) as setting parameters, we chose to use HR as it’s a classical parameter used to calibrate exercise intensity. Indeed, physical activity programs are often prescribed based on HR as it appears as the easiest objective measurement to ensure exercise intensity progression and adaptation. Aquatic exercise is known to induce physiological adaptations such as decreasing HR, depending on the level of immersion (i.e., hydrostatic pressure). All the subjects of our study were immersed between hip and umbilicus level depending on body length, which did not affect HR, as previously shown [19,20,21]. For the absolute session comparison, we chose to set the absolute intensity using the rpm, as the commercial aquacycling device does not possess a braking system to adjust the mechanical load, as the land device does. 

Our results demonstrate that, when comparing the same exercise (i.e., similar device, duration of exercise and rpm) between aquatic and land environment, water immersion induces a significant increase in EE. Because of the need to overcome drag forces induced by water, this result was not surprising. In addition, this is in line with the results of Brechat et al. [4] who suggested that aquatic cycling performed at the same workload as land-based cycling leads to a 25% increase in oxygen consumption. Another mechanism that can explain increase in EE during immersed cycling could be the heat loss, which is higher in water than in land, but we did not measure this parameter and could not confirm the hypothesis.

Regarding substrate oxidation during the different exercise sessions, results showed that the WAT session induced a higher carbohydrate oxidation than the LAND session, as also reflected by the greater RER. This is explained by the higher relative intensity of the WAT compared with the LAND session (55% of theoretical HR_max_ vs. 45%, respectively). Despite a significant difference in EE there was no difference in absolute and relative EI between sessions. The relationship between EE and EI has been shown to depend predominantly on exercise intensity [22]. High-intensity exercise (≥70% VO_2max_) has been shown to decrease REI, hunger rating and increase volitional onset of eating [22,23]. However, the pattern of eating behavior seems to be sex-specific, with appetite sensations being decreased in men [22] in response to strenuous exercise whereas women do not show these modifications in appetite sensations [24]. In the present study, we did not find any modification of appetite sensations between sessions, which is in accordance with previous studies in women [17,18,25]. Even for high-intensity exercise (e.g., ≥75% VO_2max_), previous studies did not observe any difference in appetite sensations despite a decrease in food intake [17]. In women, after an acute bout of exercise (mainly cycling), food intake modifications have been shown to depend on the weight status [26], exercise intensity [17] and cognitive restraint trait [27]. Kissileff et al. [26] have shown that a strenuous bout of cycling can induce a decrease in food intake in normal-weight women, but not in women with obesity. While we only included normal-weight women; we did not find any association between anthropometric parameters or body composition and food intake or appetite sensations. It is possible that the homogeneous population of the present study in regards to baseline characteristics does not allow for deciphering individual differences in EI and appetite sensation responses. Furthermore, the exercise intensity during the WAT session (55% of maximal HR) was probably not intense enough to affect EI. Indeed, while high-intensity exercise (>70% VO_2max_) appears to significantly impact on EI, intensities below 64% of maximal HR are considered as light (i.e., <45% of VO_2max_) [28]. Future studies on the effect of exercise intensity during immersed cycling on EI are therefore warranted.

We also investigated substrate utilization and food intake responses in an isoenergetic land compared with WAT session, matched for mean HR. Despite the two sessions being matched for EE, the WAT session induced greater carbohydrate oxidation than LAND-Iso, but this was not sufficient to create a significant difference in RER. Blood glucose levels, at the end of each session, did not show any significant difference, but marginally lower values were observed in response to the WAT session, which may be associated with the higher carbohydrate oxidation rates during this session. Kurobe et al. [29] have shown that aquatic exercises could improve glucose uptake compared with land exercises. It should be noted that the authors asked participants to ingest a glucose load before exercise, to investigate which exercise modality was more efficient to decrease postprandial glucose level during exercise. While aerobic aquatic cycling appears as a promising modality to lower postprandial glucose concentrations, effects seem to be modulated, in part, by the nutritional status of the participants (i.e., exercising 30 min after a glucose load vs. exercising 3 h after a standardized breakfast). Kurobe and colleagues also found that their aquatic session was also more efficient to increase lipolysis. Those results are consistent with the high level of carbohydrate and lipid oxidation measured in our WAT session. Physiological mechanisms explaining a specific effect of water exercise on substrate mobilization and/or oxidation are not clear. Body temperature and catecholamine release have been shown to be higher on land than in water [30] and could be potential mechanisms explaining specific effects of water on substrate utilization. We however did not measure catecholamine concentrations and did not find any difference in body temperature at the end of exercise between conditions and are thus enable to confirm these assumptions. 

Interestingly, approximately fourteen additional minutes were needed during the LAND-Iso session, matched for mean HR, to reach similar EE to that achieved in the WAT session. The significant difference in oxygen consumption, and thus metabolic power, during the two sessions could likely explain this difference. For a lower metabolic output, more time is needed to reach similar EE. This result suggests that when using HR to set exercise intensity, shorter aqua-cycling sessions can be used to induce a similar EE compared to land-based exercise sessions, without any difference in perceived exertion at the end of exercise. Aqua-cycling appears thus as a relevant new exercise training strategy in weight management, specifically for individuals with lower physical fitness (e.g., sedentary subjects and/or those with chronic diseases). We did not see a significant difference between acute exercise conditions in regards to REI, but this initial study shows promise for inducing an energy deficit (−23% relative to CONT) via water-based cycling. Future studies should examine the potential for chronic aqua-cycling programs to leverage these acute energy deficits to promote long-term negative energy balance.

We have to note several limitations in our study. Although our design and methods are in line with the habitual conditions used in aquafitness centers (especially when it comes to the methods used to calibrate the exercises), it would have been great to also perform VO_2max_ tests to better calibrate our cycling sessions in terms of intensities. As previously specified, we effectively used HR as it remains easier in free-living conditions and used by most customers during exercise training. We also chose to set the water and the land sessions at a cadence of 50 rpm because it has been identified as more comfortable in water for nonathlete subjects [16], however this cadence is considered as low for land exercise and usually 65–70 rpm is used. The same device was used for all the sessions since it was easy to use in water, but it induced a high motion rate during the LAND-Iso session (at higher rpm) which could have caused discomfort in some subjects. Finally, Edholm et al. [31] found a correlation between EE and EI two days after exercise. We did not investigate EE and EI during the evening, the day after, or two to three days after our different sessions due to practical reasons. Thus, we cannot exclude that there could have been a delayed effect of exercise on EI at dinner or during subsequent days. Further studies are thus warranted to investigate all of these factors and promote appropriate water-cycling programs.

## 5. Conclusions

The present work suggests that in healthy young women, 30 min of aqua-cycling induced similar EE to 43.6 min of land-based cycling matched for HR, without any difference in perceived exertion and REI. Thus, water cycling could represent a relevant exercise training approach, which could help in weight maintenance and weight loss strategies. Other water-based cycling modalities need to be investigated to determine how to most effectively increase EE and improve subsequent eating behavior. 

## Figures and Tables

**Table 1:** The characteristics of the subjects.

Subjects’ Characteristics	(*n* = 20)
Age (years)	27.30 ± 3.91
Weight (kg)	61.95 ± 8.44
Height (cm)	166 ± 0.06
BMI (kg.m^−2^)	22.59 ± 2.48
Waist circumference (cm)	76.92 ± 8.76
Hip circumference (cm)	89.95 ± 7.28
WHR	0.85 ± 0.04
Fat Free Mass (kg)	44.74 ± 4.48
Fat mass (kg)	16.26 ± 4.81
% Fat	25.56 ± 4.84

BMI: body mass index; WHR: waist to hip ratio.

**Table 2:** Cardiorespiratory parameters, perceived exertion and session duration, appetite feelings.

	CONT	WAT	LAND	LAND-Iso	ANOVA
**Cardiorespiratory and metabolic parameters**					
VO2 (mL·min^−1^·kg^−1^)	3.75 ± 0.75	15.24 ± 2.48 *	9.4 ± 2.2 *^,#^	12.5 ± 5 *^,#,¥^	*p* ≤ 0.001
VE (L·min)	7.5 ± 0.24	24.54 ± 1.13 *	14.27 ± 0.82 *^,#^	19.5 ± 1.07 *^,#,¥^	*p* ≤ 0.001
Mean HR (bpm)	67.6 ± 9.3	105.4 ± 11.4 *	88.9 ± 9.3 *^,#^	105.2 ± 8.2 *^,¥^	*p* ≤ 0.001
RPE End session		10.6 ± 2.7	9 ± 2.1	10.8 ± 3.2	0.52
Duration session (min)	30	30	30	43.6 ± 9.6	*p* ≤ 0.001
**Appetite feelings**					
AUC Satiety	24,156 ± 1874	27,233 ± 1730	24,000 ± 1699	26,085 ± 1463	0.85
AUC Hunger	14,530 ± 1615	13,080 ± 1180	15,390 ± 1109	14,292 ± 1268	0.78
AUC Desire to eat	15,685 ± 1522	13,907 ± 1398	15,861 ± 1127	15,342 ± 1459	0.64
AUC Prospective food consumption	16,502 ± 1402	14,882 ± 1261	16,982 ± 1185	14,623 ± 1381	0.81
SQ Satiety	−15.6 ± 2.5	−14.8 ± 4.7	−16.1 ± 7.5	−16.3 ± 5.9	0.34
SQ Hunger	17.5 ± 6.3	18 ± 8.3	17.4 ± 4.6	18.3 ± 6.4	0.52

Data are presented as mean ± SD, *n* = 20 for each condition. One –way ANOVA shows significant differences for effects session (S) for relative oxygen consumption (VO2), ventilation (VE), heart rate (HR) and duration of each session. Area under the Curve (AUC) and Satiety Quotient (SQ) for satiety and hunger are presented. Post-hoc analysis * significantly different from CONT at *p* < 0.05, ^#^ significantly different from WAT at *p* < 0.05, ^¥^ significantly different from LAND at *p* < 0.05; NS: non significant.

**Table 3:** Blood parameters and tympanic temperature during sessions and recovery.

Parameters	CONT	WAT	LAND	LAND-Iso	ANOVA
Glycemia g·L^−1^					Time effect ES:0.7 *p* = 0.03
T0	90.7 ± 8.4	92.8 ± 7.9	89.6 ± 8.9	93.3 ± 10	
T15 min	91.4 ± 5.8 *	88.3 ± 7.6 *	90.9 ± 8.4 *	92.3 ± 7.4 *	
End Exo	90.9 ± 10.1	86.4 ± 6.7	91 ± 8.5	92.4 ± 7.2	
15 min rec	88.9 ± 9.5	89.2 ± 7.3	90 ± 9.6	89.3 ± 6.5	
30 min rec	87.6 ± 7.6 *	88.3 ± 9.9 *	91 ± 10.7 *	90.7 ± 7.7 *	
Lactatemia mmol·L^−1^					ES:0.01 *p* = 0.63
T0	1.9 ± 0.4	1.8 ± 0.7	1.8 ± 0.6	1.8 ± 0.7	
T15min	1.6 ± 0.5	1.8 ± 0.6	2 ± 0.6	2 ± 0.8	
End Exo	1.5 ± 0.4	1.5 ± 0.5	1.6 ± 0.7	2 ± 0.9	
15 min rec	1.8 ± 0.7	1.3 ± 0.4	1.7 ± 1	1.6 ± 0.6	
30 min rec	1.8 ± 0.7	1.3 ± 0.2	1.7 ± 0.8	1.9 ± 1.2	
Tympanic T °C					Time effect ES: 0.8 *p* = 0.04
T0	36.8 ± 0.4	37 ± 0.4	36.9 ± 0.4	37 ± 0.7	
T15 min	36.9 ± 0.4 *	37.2 ± 0.3 *	37.1 ± 0.5 *	37.3 ± 0.4 *	
End Exo	36.8 ± 0.3	37.3 ± 0.4 *	37 ± 0.4	37.1 ± 0.3	
15 min rec	36.8 ± 0.3	36.8 ± 0.3	36.8 ± 0.3	36.8 ± 0.2	
30 min rec	36.8 ± 0.3	36.8 ± 0.4	36.8 ± 0.3	36.9 ± 0.3	

Data are presented as mean ± SD. Repeated measure ANOVA shows significant time effect (T) for glycemia and tympanic temperature (T°). Significant difference is set at *p* < 0.05; NS: non significant. Scheffe Post-hoc analysis * different from T0.

## Data Availability

The data are not publicly available due to French ethical rules for protection concerning clinical data.

## References

[1] Lee I.M., Shiroma E.J., Lobelo F., Puska P., Blair S.N., Katzmarzyk P.T., Lancet Physical Activity Series Working Group (2012). Effect of physical inactivity on major non-communicable diseases worldwide: An analysis of burden of disease and life expectancy. Lancet.

[2] Foright R.M., Presby D.M., Sherk V.D., Kahn D., Checkley L.A., Giles E.D., Bergouignan A., Higgins J.A., Jackman M.R., Hill J.O. (2018). Is regular exercise an effective strategy for weight loss maintenance?. Physiol. Behav..

[3] Rewald S., Mesters I., Lenssen A.F., Bansi J., Lambeck J., de Bie R.A., Waller B. (2017). Aquatic cycling-What do we know? A scoping review on head-out aquatic cycling. PLoS ONE.

[4] Bréchat P.H., Wolf J.P., Simon-Rigaud M.L., Bréchat N., Kantelip J.P., Berthelay S., Regnard J. (1999). Influence of immersion on respiratory requirements during 30-min cycling exercise. Eur. Respir. J..

[5] Blundell J.E., Gibbons C., Caudwell P., Finlayson G., Hopkins M. (2015). Appetite control and energy balance: Impact of exercise. Obes. Rev..

[6] Donnelly J.E., Herrmann S.D., Lambourne K., Szabo A.N., Honas J.J., Washburn R.A. (2014). Does increased exercise or physical activity alter ad-libitum daily energy intake or macronutrient composition in healthy adults? A systematic review. PLoS ONE.

[7] White L.J., Dressendorfer R.H., Holland E., McCoy S.C., Ferguson M.A. (2005). Increased caloric intake soon after exercise in cold water. Int. J. Sport Nutr. Exerc. Metab..

[8] Ueda S.Y., Nakahara H., Kawai E., Usui T., Tsuji S., Miyamoto T. (2018). Effects of walking in water on gut hormone concentrations and appetite: Comparison with walking on land. Endocr. Connect..

[9] Thackray A.E., Willis S.A., Sherry A.P., Clayton D.J., Broom D.R., Demashkieh M., Sargeant J.A., James L.J., Finlayson G., Stensel D.J. (2020). An acute bout of swimming increases post-exercise energy intake in young healthy men and women. Appetite.

[10] Isacco L., Duché P., Boisseau N. (2012). Influence of hormonal status on substrate utilization at rest and during exercise in the female population. Sports Med..

[11] Thivel D., Genin P.M., Mathieu M.E., Pereira B., Metz L. (2016). Reproducibility of an in-laboratory test meal to assess ad libitum energy intake in adolescents with obesity. Appetite.

[12] Flint A., Raben A., Blundell J.E., Astrup A. (2000). Reproducibility, power and validity of visual analogue scales in assessment of appetite sensations in single test meal studies. Int. J. Obes. Relat. Metab. Disord..

[13] Drapeau V., King N., Hetherington M., Doucet E., Blundell J., Tremblay A. (2007). Appetite sensations and satiety quotient: Predictors of energy intake and weight loss. Appetite.

[14] Péronnet F., Massicotte D. (1991). Table of nonprotein respiratory quotient: An update. Can. J. Sport Sci..

[15] Borg G., Hassmen P., Lagerstrom M. (1987). Perceived exertion related to heart rate and blood lactate during arm and leg exercise. Eur. J. Appl. Physiol. Occup. Physiol..

[16] Hall J., Blake D., Garbutt G. (2001). Acute physiological effects of exercise in water. Phys. Ther. Rev..

[17] Pomerleau M., Imbeault P., Parker T., Doucet E. (2004). Effects of exercise intensity on food intake and appetite in women. Am. J. Clin. Nutr..

[18] Rocha J., Paxman J., Dalton C., Winter E., Broom D. (2015). Effects of an acute bout of aerobic exercise on immediate and subsequent three-day food intake and energy expenditure in active and inactive pre-menopausal women taking oral contraceptives. Appetite.

[19] Sheldahl L.M., Tristani F.E., Clifford P.S., Hughes C.V., Sobocinski K.A., Morris R.D. (1987). Effect of head-out water immersion on cardiorespiratory response to dynamic exercise. J. Am. Coll. Cardiol..

[20] Christie J.L., Sheldahl L.M., Tristani F.E., Wann L.S., Sagar K.B., Levandoski S.G., Ptacin M.J., Sobocinski K.A., Morris R.D. (1990). Cardiovascular regulation during head-out water immersion exercise. J. Appl. Physiol. (1985).

[21] Garzon M., Juneau M., Dupuy O., Nigam A., Bosquet L., Comtois A., Gayda M. (2015). Cardiovascular and hemodynamic responses on dryland vs. immersed cycling. J. Sci. Med. Sport.

[22] King N.A., Burley V.J., Blundell J.E. (1994). Exercise-induced suppression of appetite: Effects on food intake and implications for energy balance. Eur. J. Clin. Nutr..

[23] Charlot K., Chapelot D. (2019). Comparison of energy-matched high-intensity interval and moderate-intensity continuous exercise sessions on latency to eat, energy intake, and appetite. Appl. Physiol. Nutr. Metab..

[24] King N.A., Snell L., Smith R.D., Blundell J.E. (1996). Effects of short-term exercise on appetite responses in unrestrained females. Eur. J. Clin. Nutr..

[25] Rocha J., Paxman J.R., Dalton C.F., Hopkins M., Broom D.R. (2018). An acute bout of cycling does not induce compensatory responses in pre-menopausal women not using hormonal contraceptives. Appetite.

[26] Kissileff H.R., Pi-Sunyer F.X., Segal K., Meltzer S., Foelsch P.A. (1990). Acute effects of exercise on food intake in obese and nonobese women. Am. J. Clin. Nutr..

[27] Lluch A., King N.A., Blundell J.E. (2000). No energy compensation at the meal following exercise in dietary restrained and unrestrained women. Br. J. Nutr..

[28] Garber C.E., Blissmer B., Deschenes M.R., Franklin B.A., Lamonte M.J., Lee I.M., Nieman D.C., Swain D.P., Medicine A.C.o.S. (2011). American College of Sports Medicine position stand. Quantity and quality of exercise for developing and maintaining cardiorespiratory, musculoskeletal, and neuromotor fitness in apparently healthy adults: Guidance for prescribing exercise. Med. Sci. Sports Exerc..

[29] Kurobe K., Kousaka A., Ogita F., Matsumoto N. (2018). Metabolic responses to exercise on land and in water following glucose ingestion. Clin. Physiol. Funct. Imaging.

[30] Fujishima K., Shimizu T., Ogaki T., Hotta N., Kanaya S., Shono T., Ueda T. (2001). Thermoregulatory responses to low-intensity prolonged swimming in water at various temperatures and treadmill walking on land. J. Physiol. Anthropol. Appl. Hum. Sci..

[31] Edholm O.G., Fletcher J.G., Widdowson E.M., McCance R.A. (1955). The energy expenditure and food intake of individual men. Br. J. Nutr..

